# The Role of Prognostic Nutritional Index in Predicting Coronary Slow Flow Phenomena

**DOI:** 10.3390/diagnostics15182324

**Published:** 2025-09-13

**Authors:** Murat Özmen, Onur Altınkaya, Selim Aydemir, Sidar Şiyar Aydın, Faruk Aydınyılmaz

**Affiliations:** 1 Department of Cardiology, University of Health Sciences, Erzurum Bolge Training and Research Hospital, 25240 Erzurum, Turkey; onuraltinkaya35@gmail.com (O.A.); selim1723@hotmail.com (S.A.); faruk_aydinyilmaz@hotmail.com (F.A.); 2 Department of Cardiology, Ataturk University, 25040 Erzurum, Turkey; s.siyaraydin@gmail.com

**Keywords:** coronary slow flow, prognostic nutritional index, coronary angiography

## Abstract

**Background/Objectives:** Coronary slow flow phenomenon (SSF) is a syndrome defined by the filling of low-velocity contrast material into the distal portion of one or more coronary arteries despite the absence of coronary artery stenosis on coronary angiography (CAG). The exact cause of this condition has not yet been determined. In our study, we planned to investigate the relationship between prognostic nutritional index (PNI) and BOS. **Methods:** In total, 2585 patients who underwent coronary angiography between October 2021 and October 2023 were included in the study. In total, 181 patients with BOS and available serum albumin and lymphocyte data were evaluated. **Results:** The mean age of the study group was 58 ± 11 years. Specifically, in univariate and multivariate regression analyses, age, diabetes mellitus (DM), hemoglobin (Hb), and PNI were found to be statistically significant in predicting BOS. In ROC analysis, the cut-off value for PNI was 39.01, with 76.2% sensitivity and 76.2% specificity (AUC: 0.788; 95% CI: 0.749–0.824) in predicting CSF. **Conclusions:** Our study demonstrated that PNI, an easily measured, accessible, and inexpensive parameter that reflects inflammation and nutritional status, can be used in predicting CSF.

## 1. Introduction

Coronary slow flow is characterized as a microvascular disorder consisting of the slow progression of the opaque material administered during coronary angiography to the distal vascular bed without occlusion of the epicardial coronary arteries. This is a significant clinical condition that can cause chest pain at rest or on exercise [1]. CSF is not an uncommon occurrence in patients undergoing routine coronary angiography. In one study, CSF was seen in 1% of patients who underwent coronary angiography, while in another study, this rate was 7% [2,3]. In CSF patients, hypertension was detected in 58%, smoking in 80%, family history in 45%, dyslipidemia in 45%, and diabetes mellitus (DM) in 22% [4]. Today, the pathophysiological mechanisms underlying CSF are still not fully understood. Most authors have stated that CSF should be considered as a clinical syndrome rather than an angiographic phenomenon [5,6]. However, many questions remain about whether this pathology is limited to the coronary arteries or whether it is a manifestation of systemic vascular or endothelial disease. Hypotheses and theories about the mechanism of CSF include endothelial dysfunction, increased vasomotor tone, small vessel disease, inflammatory phenomena, and widespread atherosclerosis [6,7,8]. In these patients, the epicardial major coronary arteries are normal, but the coronary arteries (conducting vessels) that regulate normal antegrade blood flow to accommodate increased myocardial oxygen demand are reduced. This decreased blood flow, combined with endothelial dysfunction, leads to increased microvascular resistance, resulting in myocardial ischemia, which can lead to systolic and diastolic dysfunction [6,7].

Prognostic nutritional index (PNI) was first used in 1980 by Buzby et al. and defined by Onodera et al. [9,10] PNI is an index calculated based on serum albumin concentration and lymphocyte count. Today, it has been shown that PNI may be associated with prognosis in many diseases [11,12]. The usefulness of PNI as a prognostic indicator has been shown in various diseases such as gastrointestinal cancer [13], lymphoma [14], hip fractures [15], ischemic stroke [16,17]. In the studies conducted by T. Narumi and colleagues, PNI has prognostic importance in patients with heart failure [11]. In another study, PNI was found to be associated with cardiovascular events and increased mortality in cases of acute coronary syndrome [13].

Calculated based on serum albumin and lymphocyte counts, the PNI serves as an immunonutritional marker that reflects the body′s chronic inflammation, immune function, and nutritional status [14]. Considering the relationship between inflammatory and nutritional status and CSF, PNI showing these parameters may be related to CSF.

CSF is widely believed to be a benign condition; however, recurrent angina pectoris, recurrences, ventricular arrhythmias, hospitalization, and even sudden cardiac death have been reported in this patient group [18]. It has been suggested that it may cause myocardial infarction in patients with normal accompanying angiography results [18]. There is a paucity of data and conflicting reports regarding the relationship between BOS and patient prognosis, particularly the history and causes of coronary events.

In this retrospective observational study, we aimed to demonstrate the relationship between PNI, a cost-effective score calculated solely from blood parameters and used as an indicator of malnutrition, a significant health problem, and coronary slow flow.

## 2. Materials and Methods

### 2.1. Study Population

**Figure 1:**
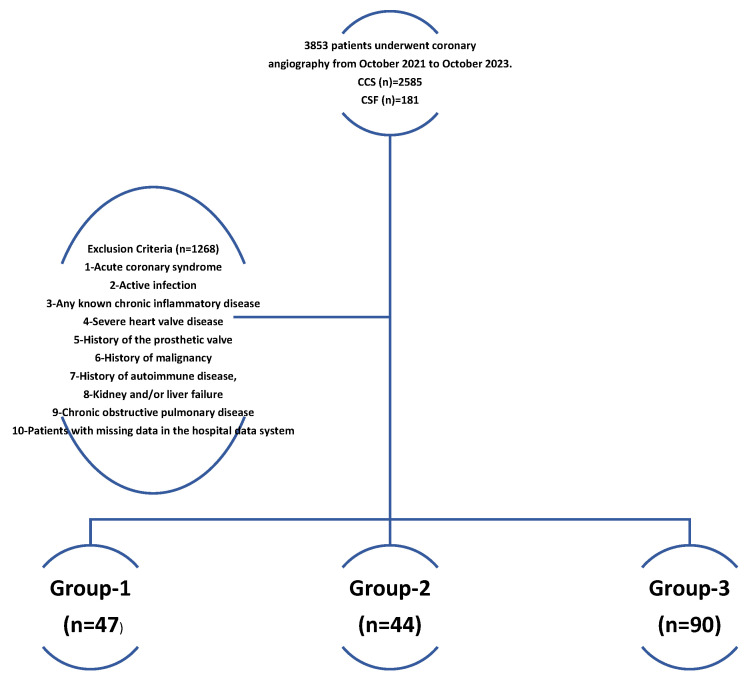
A flow chart of the patients included in the study. Group-1 = PNI value > 38, Group-2 PNI value between 35 and 38, Group-3 = PNI value < 35), (PNI: prognostic nutritional index; CSF: Coronary slow flow phenomenon).

A total of 3853 patients who underwent coronary angiography at Erzurum City Hospital, Türkiye, between October 2021 and October 2023 were evaluated. Of these, 2585 were diagnosed with chronic coronary syndrome (CCS). Patients with acute coronary syndrome, active infection, known history of chronic inflammatory disease, severe heart valve disease, history of malignancy, history of prosthetic valves, renal and/or liver failure, history of autoimmune disease, chronic obstructive pulmonary disease, and missing data in the hospital data system were excluded from the study. Patients were divided into two groups: those with CSF (*n* = 181) and those without CSF (*n* = 2414). The PNI values of all patients were calculated, and the difference between the two groups was evaluated (Figure 1).

Our study was approved by the University of Health Sciences Erzurum Faculty of Medicine Scientific Research Ethics Committee (Date: 11 October 2023; Decision number: 2023/06-68). It was conducted in accordance with the Declaration of Helsinki.

### 2.2. Coronary Angiography

Patients in whom coronary angiography was performed using the Judkins technique via radial and femoral interventions according to standard protocols in the catheterization department of our hospital were included in the study. The Siemens (Erlanger, Germany) brand angiography device was used to visualize the coronary arteries. Iohexol (KOPAQ 350 mg/mL) was used as a radiopaque material in the angiographies. All CAG evaluations were performed independently by two experienced invasive cardiologists who were unaware of the patients′ clinical details. The thrombolysis frame count (TFC) method was used for quantitative assessment of coronary blood flow in myocardial infarction as described in the literature. The time it took for the contrast to reach distant points of the coronary artery was defined as the number of frames. The onset is when the contrast travels through the artery and begins to contact both sides. As an endpoint, the distal bifurcation of the most extended branch for the circumflex (CX) is visualized when the contrast agent arrives at the distal branch point called the whale′s tail region for the left anterior descending (LAD) and gives off the first lateral branch of the posterolateral artery for the right coronary artery (RCA). Because the LAD has a prolonged course compared to other arteries, the value was standardized by dividing it by 1.7. The number of frames made by shooting at 12 fps was multiplied by 2.4, a fixed number since former studies shot at 30 fps. When the sum of the three vessels divided by 3, the mean TFC was acquired. The cut off value of TFCs were 36.2 ± 2.6 for the LAD (cTFC 21.1 ± 1.5), 22.2 ± 4.1 for the LCX, and 20.4 ± 3 for the RCA [19,20]. The patients did not have severe epicardial stenosis that would prevent TIMI-3 flow.

### 2.3. Laboratory Analysis

Patients with blood parameters recorded in the hospital data system were included in the study. Hemogram values were studied with the Sysmex XN-1000 Clinical System. Biochemical parameters were studied with the BECKMAN COULTER AV 5800 Clinical System. The PNI was calculated with the formula PNI = 10 × serum albumin (g/dL) + 0.005 × total lymphocyte count (/mm^3^) [10].

### 2.4. Statistical Analysis

Statistical analysis was performed using IBM SPSS Statistics 27.0 for Windows. The Kolmogorov–Smirnov test was used to examine the normal distribution of the data. Continuous variables with normal distribution were shown as mean ± standard deviation, and those without normal distribution were shown as median and interquartile range values. Categorical data were expressed as numbers and percentages. Student-t test or Mann–Whitney U test was used for numerical variables, and chi-square test was used in the analysis of categorical variables. The relationship between two independent numerical variables was evaluated using Spearman and Pearson correlation coefficients. Parametric variables belonging to three independent groups were assessed with ANOVA, an appropriate chi-square test was used to evaluate categorical variables, and the results were considered statistically significant when the *p*-value < 0.05. Post hoc Tukey analysis was performed to investigate the significance between groups in three-way comparisons. ROC curve analysis was performed to see the relationship between CSF and PNI in the whole group. In the ROC analysis, the cut-off value was calculated according to the Youden index. To prevent visual misunderstandings, 1/PNI was used.

## 3. Results

In this study, 2585 patients who underwent coronary angiography were screened, and CSF was detected in 181 patients. Demographic characteristics of patients with and without CSF are displayed in Table 1. The ages of patients with and without CSF were statistically significant (58 ± 11 and 62 ± 12, *p* < 0.001, respectively). While CAD, AF, and HT were not statistically significant, DM was statistically significant (*p* = 0.54, *p* = 0.13, *p* = 0.55; *p* = 0.003). Hb, platelet (PLT), albumin, triglyceride, and PNI were significant, while other laboratory parameters were not statistically significant. There was no difference in the drug composition for β-blockers, long-acting nitrates, calcium-channel blockers, lipid-lowering drugs, angiotensin-converting enzyme (ACE) inhibitors, angiotensin receptor blockers (ARB), acetylsalicylic acid, and oral anticoagulants (*p* > 0.05) (Table 1).

Univariate and multivariate regression analysis was performed between the parameters significantly associated with CSF. In univariate and multivariate regression analyses, age, DM, triglyceride, Hb, and PNI were observed to be statistically significant (Table 2).

Data on patients with the coronary slow flow phenomenon, classified according to PNI values, are shown in Table 3. Male gender comes to the fore, especially in patients with CSF. In univariate and multivariate regression analysis, age, DM, hemoglobin, triglyceride, and PNI were observed to be statistically significant (Table 2). Patients with CSF were separated into three groups according to their PNI values. Those with a PNI value > 38 were called group-1, those with a PNI value between 35 and 38 were called group-2, and those with a PNI value < 35 were called group-3 [11]. No significant statistical difference was observed between the groups in terms of gender, C. A statistically significant difference was observed between groups in DM, albumin, and PNI values. (*p* < 0.001) (Table 3).

No significant statistical difference was observed between the three groups in terms of age and gender (*p* > 0.05). CAD was more common in Group 3 patients (91.1%). AF was more common in Group 2 patients (18.2%). DM was more common in Group 1 patients (21.3%). HT was observed more commonly in group 3 patients, with a rate of 53.3%. No statistically significant difference was observed between the groups in terms of β-blockers, long-acting nitrates, calcium-channel blockers, lipid-lowering drugs, angiotensin-converting enzyme (ACE) inhibitors, angiotensin receptor blockers (ARB), acetylsalicylic acid, and oral anticoagulant drugs (*p* > 0.05) (Table 3).

**Figure 2:**
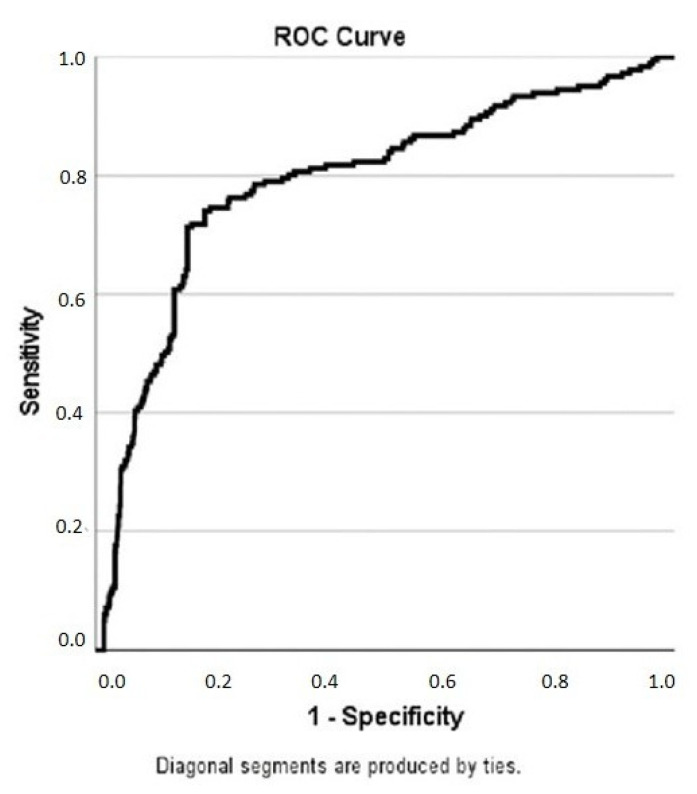
ROC analysis of the relationship between PNI value and CSF. To avoid visual misunderstanding, 1/PNI was used.

ROC Curve analysis was performed to determine the predictive power of PNI, which independently predicts CSF in regression analysis, and the sensitivity and specificity of the cut-off value at this value. In the ROC analysis, the cut-off value for PNI was 39.01, with 76.2% sensitivity and 76.2% specificity (AUC: 0.788; 95% CI: 0.749–0.827) in predicting CSF (Figure 2).

## 4. Discussion

This study examines the correlation between nutritional status and the development of coronary slow flow. Our results showed a relationship between low PNI values and CSF.

Opacity of the distal vessels, known as cardiac syndrome Y, occurs when the coronary arteries are normal [21]. It has clinical effects such as angina, acute coronary syndrome, and sudden death [22]. CSF is defined as delayed opacification of the coronary arteries at distal levels in the absence of occluded vessels. This condition results from microvascular dysfunction. In some studies, it has been observed as an early sign of atherosclerotic disease [23,24]. CSF was first described in 6 patients and was later described by Tambe et al. [25]. In a study, increased coronary vasomotor tone and impaired endothelial dilatation in coronary resistance vessels came to the fore [26]. Some studies have shown that higher NO synthase levels, together with lower nitric oxide (NO) and impaired endothelial function, may contribute to this pathomechanism [27]. In CSF, all three coronary arteries may be affected, resulting in a more severe and widespread condition, which indicates a poorer prognosis [28]. Several pathomechanisms have been proposed, particularly in the formation of CSF. These include impaired endothelium-dependent flow-mediated dilatation [15,16], microvascular spasm [17], increased resistance in the epicardial coronary arteries due to widespread atherosclerosis resulting from decreased fractional flow reserve [19], and inflammatory phenomena and conditions that may be associated with CSF [15,16]. In various previous studies, PNI is related to different inflammatory processes [17].

Inflammation plays a crucial role in the progression of atherosclerosis. However, inflammation causes endothelial dysfunction. Plays an important role in the initiation and progression of CSF. CRP and albumin, which are markers of inflammation, are biochemical parameters routinely used in clinical practice. There is a significant relationship between increased CRP and the severity of stable angina pectoris, myocardial infarction, stroke, and coronary artery disease (CAD) [29]. Albumin is a negative acute phase reactant. A decrease in albumin levels leads to endothelial dysfunction and a rise in inflammatory cytokines. This is important for the development of cardiovascular disease [30]. In our study, albumin level was lower in PNI group-3 patients.

A few studies have examined coronary blood flow in normal coronary arteries without categorizing diabetes separately, and the results of these studies have mainly been conflicting [31]. In CSF syndrome, it has been suggested that insulin resistance may lead to the development of coronary microvascular dysfunction before the development of overt diabetes [32]. The higher CSF observed in the non-DM group in our study may be explained by the fact that the patients had not yet developed diabetes despite having developed insulin resistance and microvascular dysfunction, and that the relatively younger patients had non-obstructive CSF. In our current study, the patients with CSF were followed up at a younger age. This supports studies in the literature [6,33,34].

While there is no definitive treatment protocol for patients with slow coronary flow, acetylsalicylic acid, angiotensin-converting enzyme inhibitors, and statins may be administered to improve endothelial function. The value of conventional antianginal therapies in these patients is limited. Additionally, dipyridamole and calcium channel blockers are accepted agents for the treatment of slow coronary flow [35]. In a study conducted with atorvastatin, they demonstrated that short-term lipid-lowering therapy improved coronary flow reserve, an indicator of coronary microvascular function, in patients with CSF [36]. Another study conducted with simvastatin demonstrated that simvastatin improved myocardial perfusion in patients with CSF [37]. Our study supports these studies and demonstrated statistical significance in patients without CSF.

A parameter that can evaluate all aspects of nutritional status has yet to be developed. Formulas such as the PNI and nutritional risk index (NRI) have been put forward for this purpose [9]. PNI is a combined index that reflects patients′ immunological and nutritional status. PNI is calculated using albumin and lymphocyte values from peripheral venous blood samples taken from patients. Again, PNI has been previously found to be associated with prognosis in patients with congestive heart failure, ST-elevation myocardial infarction, and acute coronary syndrome, which are accompanied by inflammation and have similar inflammatory pathophysiology [11]. Our study supports existing studies and shows statistical significance between CSF, a predictor of acute coronary syndrome, and PNI. Although weight loss is a predictor of mortality in patients with ACS and heart failure, the relationship between nutritional status and cardiovascular disease has not been fully established [38]. However, studies conducted with PNI, one of the nutritional indices, have shown that patients with lower PNI tend to have more than one comorbidity. Our study supports existing studies and shows that patients with lower PNI values have more than one comorbidity. Another study found that low PNI predicted adverse outcomes in stable angina pectoris [39]. Our study observed statistical significance between CSF and PNI, a predictor of angina.

CSF should not be considered a benign condition and should be detected immediately, as previous studies have shown that CSF has prognostic significance in the development of major adverse cardiovascular events [40,41]. Coronary angiography is the gold standard for CSF identification. However, it is not easily accessible in developing countries because it is an invasive procedure. In long-term follow-up, it is challenging to evaluate the benefits of coronary angiography due to its associated costs. Therefore, well-performing and cost-effective parameters can be integrated into the clinic. A cheap, easily calculated, and practical parameter such as PNI, which can be used in routine tests, can contribute to early risk stratification, treatment adjustment, prognostication, and the development of new treatment modalities.

Our study has several limitations. First, it is a single-center, retrospective study. Our study included a relatively small number of CSF patient populations and large-scale studies are needed. In the second study, BOS was diagnosed by visual assessment of CAG, which does not provide sufficient information about true coronary blood flow. Coronary endothelial function was not assessed using intravascular ultrasound or combined pressure and flow studies in our study. Furthermore, body mass index data were not available. Data on medical treatment administered to patients after CSF was not available due to the retrospective nature of our study. Further, larger, multicenter prospective studies are needed to confirm our findings.

## 5. Conclusions

This study demonstrated that PNI, an easily measured, accessible, and inexpensive parameter, is associated with CSF and can be used as a predictive parameter for CSF. PNI′s ability to predict CSF, a condition that may predispose to coronary atherosclerosis and acute coronary syndrome, may be beneficial for early risk stratification, treatment planning, and prognosis. Future prospective studies confirming the relationship between PNI and CSF in different populations may contribute to the pathogenesis and treatment of CSF.

## Figures and Tables

**Table 1:** Characteristics of demographic and clinical data.

	CSF(+) (*n* = 181)	CSF(−) (*n* = 2404)	*p* Value
Gender (M) *n* (%)	125 (69)	1544 (64)	0.11
Age	58 ± 11	62 ± 12	**<0.001**
CAD *n* (%)	160 (88.4)	2131 (88.3)	0.54
AF *n* (%)	21 (11.6)	217 (9)	0.13
DM *n* (%)	20 (11)	465 (19.3)	**0.003**
HT *n* (%)	105 (58)	1557 (64.5)	0.55
β-Blocker *n* (%)	46 (25.9)	1514 (62.1)	0.07
Long-acting nitrates *n* (%)	19 (10.4)	1298 (53.8)	0.08
Calcium-channel blockers *n* (%)	23 (12.9)	759 (31.4)	0.06
Lipid-lowering drugs *n* (%)	25 (13.8)	2123 (87.9)	**0.03**
ACE inhibitors *n* (%)	56 (30.9)	1146 (47.4)	0.06
ARB *n* (%)	49 (27)	411(17.2)	0.14
Acetylsalicylic acid *n* (%)	130 (71.8)	2123(87.9)	0.16
Oral anticoagulants *n* (%)	20 (11)	215(8.9)	0.23
HbA1c (%)	6.91 ± 1.61	5.79 ± 0.74	**0.01**
Systolic BP, mm Hg	136.7 ± 19.07	134.2 ± 16.76	0.15
Diastolic BP, mm Hg	84.96 ± 12.14	83.42 ± 10.62	0.18
Heart rate (bpm)	74.9 ± 24	76.2 ± 27	0.19
WBC 10^3^/mm^3^	10.07 ± 0.3	10.02 ± 3.7	0.84
Neutrophil 10^9^/L	6.7 ± 1.3	6.4 ± 1.4	0.25
Hb g/dL	14.4 ± 2.1	14.09 ± 2.3	**0.01**
PLT 10^3^/mm^3^	243.5 ± 67.6	251.6 ± 77.8	**0.02**
Lymphocyte 10^3^/mm^3^	2.1 ± 0.9	2.0 ± 1.0	0.22
Glukose mg/dL	133 ± 70	123 ± 62	0.061
Albumine g/dL	33.9 ± 6.7	40 ± 6.9	**<0.001**
Total Cholesterol mg/dL	169 ± 49	170 ± 48	0.97
LDL mg/dL	124 ± 39	125 ± 40	0.59
HDL mg/dL	46 ± 11	40 ± 12	0.08
Triglyceride mg/dL	170 ± 77	148 ± 58	**0.001**
Creatinine mg/dL	0.9 ± 0.7	0.8 ± 0.5	0.72
Sodium mmol/L	138 ± 3.9	139 ± 4.8	0.91
Potassium mmol/L	4.2 ± 0.4	4.3 ± 0.6	0.73
Calcium mg/dL	9.2 ± 0.4	9.3 ± 0.6	0.40
CRP mg/dL	2.8 ± 0.8	4.7 ± 2.1	0.27
Uric Asid mg/dL	5.4 ± 1.7	5.5 ± 2.1	0.23
NLR	3.9 ± 2.5	4.1 ± 3.6	0.89
CAR	0.07 ± 0.06	0.13 ± 0.18	0.57
PNI	35.0 ± 6	40.6 ± 6.6	**<0.001**

Abbreviations: CAD: Coronary Artery Disease, AF: Atrial Fibrillation, DM: Diabetes Mellitus, HT: Hypertension, ACE: Angiotensin-Converting Enzyme Inhibitors, ARB: Angiotensin Receptor Blockers, BP: Blood Pressure WBC: White Blood Count, Hb: Hemoglobin, PLT: Platelet, LDL: Low-Density Lipoprotein, HDL: High-Density Lipoprotein, CRP: C-Reactive Protein, NLR: Neutrophil Lymphocyte Ratio, CAR: C-Reactive Protein Albumin Ratio PNI: Prognostic Nutritional Index.

**Table 2:** Effects of different variables on CSF in univariate and multivariate logistic regression analysis.

	Univariate			Multivariate		
	OR	% 95 CI	*p*	OR	% 95 CI	*p*
Age	0.978	0.966–0.990	<0.001	0.977	0.964–0.991	**0.001**
DM	0.491	0.304–0.793	0.004	0.463	0.270–0.792	**0.005**
Hb (g/dL)	1.089	1.016–1.167	0.010	1.173	1.084–1.268	**<0.001**
Triglyceride mg/dL	1.004	1.002–1.006	<0.001	1.003	1.001–1.005	**0.005**
PNI	0.887	0.866–0.908	<0.001	0.878	0.855–0.901	**<0.001**

OR: Odds Ratio, CI: confidence interval, DM: Diabetes Mellitus, Hb: Hemoglobin, PNI: Prognostic Nutritional Index.

**Table 3:** Characteristics of demographic and clinical data according to PNI values.

	CSF (*n* = 181)	Group-1 (*n* = 47)	Group-2 (*n* = 44)	Group-3 (*n* = 90)	*p*
Gender (M) (%)	125 (69)	29 (63.8)	28 (65.9)	59 (66)	0.33
Age	62 ± 12	61 ± 11	62 ± 13	60 ± 10	0.11
CAD *n* (%)	160 (88.4)	41 (87.2)	37 (84.1)	81 (91.1)	0.47
AF *n* (%)	21 (11.6)	6 (12.8)	8 (18.2)	7 (7.8)	0.20
DM *n* (%)	20 (11)	10 (21.3)	5 (11.4)	5 (5.6)	**0.020 ^b^**
HT *n* (%)	105 (58)	32 (68.1)	24 (56.8)	47 (53.3)	0.25
β-Blocker *n* (%)	46 (25.9)	15 (31.9)	17 (38.6)	14 (15.5)	0.25
Long-acting nitrates *n* (%)	19 (10.4)	9 (19.1)	5 (11.3)	5 (5.5)	0.34
Calcium-channel blockers *n* (%)	23 (12.9)	7 (14.8)	10 (22.7)	6 (7)	0.89
Lipid-lowering drugs *n* (%)	25 (13.8)	8 (17)	9 (20.4)	8 (9)	0.19
ACE inhibitors *n* (%)	56 (30.9)	24 (51)	19 (43)	13 (14)	0.76
ARB *n* (%)	49 (27)	22 (46.8)	21 (47)	6 (7)	0.54
Acetylsalicylic acid *n* (%)	130 (71.8)	40 (85)	36 (82)	54 (60)	0.24
Oral anticoagulants *n* (%)	20 (11)	8 (17)	7 (16)	5 (6)	0.46
WBC 10^3^/mm^3^	10.07 ± 0.3	9.09 ± 2.5	10.6 ± 5	10.3 ± 3.4	0.098
Hb g/dL	14.4 ± 2.1	15 ± 2.1	14.2 ± 2.2	14.3 ± 2	0.099
PLT 10^3^/mm^3^	243.5 ± 67.6	251.6 ± 66.3	242.8 ± 65.6	239.6 ± 69.6	0.61
Lymphocyte 10^3^/mm^3^	2.1 ± 0.9	2.2 ± 0.8	2.1 ± 0.9	2 ± 0.8	0.93
Glukose mg/dL	133 ± 70	140 ± 86	112 ± 37	119 ± 55	0.07
Albumine g/dL	33.9 ± 6.7	43.3 ± 3.2	31.2 ± 5.4	30 ± 2.4	**<0.001 ^a,b^**
Total Cholesterol mg/dL	169 ± 49	176 ± 50	160 ± 50	168 ± 47	0.43
LDL mg/dL	124 ± 39	120 ± 40	129 ± 40	124 ± 39	0.73
HDL mg/dL	46 ± 11	47 ± 14	39 ± 6	49 ± 9	0.34
Triglyceride mg/dL	170 ± 77	150 ± 61	149 ± 73	156 ± 65	0.99
Creatinine mg/dL	0.9 ± 0.7	0.8 ± 0.2	0.8 ± 0.3	1 ± 0.9	0.42
Sodium mmol/L	138 ± 3.9	139 ± 3	138 ± 2	138 ± 4	0.72
Potassium mmol/L	4.2 ± 0.4	4.3 ± 0.6	4.1 ± 0.4	4.1 ± 0.5	0.31
Calcium mg/dL	9.2 ± 0.4	9.3 ± 0.6	9.2 ± 0.3	9.1 ± 0.6	0.30
CRP mg/dL	2.8 ± 0.8	2.5 ± 1	2.9 ± 0.9	3.1 ± 0.7	0.95
Uric Aside mg/dL	5.4 ± 1.7	5.7 ± 1.8	5.2 ± 1.6	5.2 ± 1.8	0.31
PNI	35.0 ± 6	43.4 ± 3.2	36 ± 0.7	30.2 ± 2.4	**<0.001 ^a,b,c^**

CAD: Coronary Artery Disease, AF: Atrial Fibrillation, DM: Diabetes Mellitus, HT: Hypertension, ACE: Angiotensin-Converting Enzyme Inhibitors, ARB: Angiotensin Receptor Blockers, WBC: White Blood Count, Hb: Hemoglobin, PLT: Platelet, LDL: Low-Density Lipoprotein, HDL: High-Density Lipoprotein, CRP: C-Reactive Protein, PNI: Prognostic Nutritional Index. ^a^: Group 1 and 2; ^b^: Group 1 and 3; ^c^: Group 2 and 3.

## Data Availability

The raw data supporting the conclusions of this article will be made available by the authors on request.

## Abbreviations

The following abbreviations are used in this manuscript:

CSF	Coronary Slow Flow Phenomenon
PNI	Prognostic Nutritional Index
DM	Diabetes Mellitus
CAG	Coronary Angiography
LAD	Left Anterior Descending
CX	Circumflex
RCA	Right Coronary Artery (RCA).
CAD	Coronary Artery Disease
HT	Hypertension
AF	Atrial Fibrillation

## References

[1] Xia S., Deng S.B., Wang Y., Xiao J., Du J.L., Zhang Y., Wang X.C., Li Y.Q., Zhao R., He L. (2011). Clinical analysis of the risk factors of slow coronary flow. Heart Vessel..

[2] Singh S., Kothari S.S., Bahl V.K. (2004). Coronary slow flow phenomenon: An angiographic curiosity. Indian Heart J..

[3] Oktay V., Arat Ozkan A. (2016). Coronary slow flow. Turk. Kardiyol. Dern. Ars..

[4] Yazici M., Demircan S., Aksakal E., Sahin M., Meric M., Dursun I., Yuksel S., Sagkan O. (2003). Plasma insulin, glucose and lipid levels, and their relations with corrected TIMI frame count in patients with slow coronary flow. Anadolu Kardiyol. Derg..

[5] Zavala-Alarcon E., Cecena F., Little R., Bant A., Van Poppel S., Patel R. (2005). The no-flow phenomenon during diagnostic coronary angiography. Cardiovasc. Revasc. Med..

[6] Aparicio A., Cuevas J., Moris C., Martin M. (2022). Slow Coronary Blood Flow: Pathogenesis and Clinical Implications. Eur. Cardiol..

[7] Chalikias G., Tziakas D. (2021). Slow Coronary Flow: Pathophysiology, Clinical Implications, and Therapeutic Management. Angiology.

[8] Kalay N., Aytekin M., Kaya M.G., Ozbek K., Karayakali M., Sogut E., Altunkas F., Ozturk A., Koc F. (2011). The relationship between inflammation and slow coronary flow: Increased red cell distribution width and serum uric acid levels. Turk. Kardiyol. Dern. Ars..

[9] Buzby G.P., Mullen J.L., Matthews D.C., Hobbs C.L., Rosato E.F. (1980). Prognostic nutritional index in gastrointestinal surgery. Am. J. Surg..

[10] Onodera T., Goseki N., Kosaki G. (1984). Prognostic nutritional index in gastrointestinal surgery of malnourished cancer patients. Nihon Geka Gakkai Zasshi.

[11] Narumi T., Arimoto T., Funayama A., Kadowaki S., Otaki Y., Nishiyama S., Takahashi H., Shishido T., Miyashita T., Miyamoto T. (2013). Prognostic importance of objective nutritional indexes in patients with chronic heart failure. J. Cardiol..

[12] Hayashi J., Uchida T., Ri S., Hamasaki A., Kuroda Y., Yamashita A., Sadahiro M. (2020). Clinical significance of the prognostic nutritional index in patients undergoing cardiovascular surgery. Gen. Thorac. Cardiovasc. Surg..

[13] Raposeiras Roubin S., Abu Assi E., Cespon Fernandez M., Barreiro Pardal C., Lizancos Castro A., Parada J.A., Perez D.D., Blanco Prieto S., Rossello X., Ibanez B. (2020). Prevalence and Prognostic Significance of Malnutrition in Patients With Acute Coronary Syndrome. J. Am. Coll. Cardiol..

[14] Wang D., Hu X., Xiao L., Long G., Yao L., Wang Z., Zhou L. (2021). Prognostic Nutritional Index and Systemic Immune-Inflammation Index Predict the Prognosis of Patients with HCC. J. Gastrointest. Surg..

[15] Sanati H., Kiani R., Shakerian F., Firouzi A., Zahedmehr A., Peighambari M., Shokrian L., Ashrafi P. (2016). Coronary Slow Flow Phenomenon Clinical Findings and Predictors. Res. Cardiovasc. Med..

[16] Li J.J., Qin X.W., Li Z.C., Zeng H.S., Gao Z., Xu B., Zhang C.Y., Li J. (2007). Increased plasma C-reactive protein and interleukin-6 concentrations in patients with slow coronary flow. Clin. Chim. Acta.

[17] Tokunaga R., Sakamoto Y., Nakagawa S., Miyamoto Y., Yoshida N., Oki E., Watanabe M., Baba H. (2015). Prognostic nutritional index predicts severe complications, recurrence, and poor prognosis in patients with colorectal cancer undergoing primary tumor resection. Dis. Colon Rectum.

[18] Zivanic A., Stankovic I., Ilic I., Putnikovic B., Neskovic A.N. (2020). Prognosis of patients with previous myocardial infarction, coronary slow flow, and normal coronary angiogram. Herz.

[19] Gibson C.M., Cannon C.P., Daley W.L., Dodge J.T., Alexander B., Marble S.J., McCabe C.H., Raymond L., Fortin T., Poole W.K. (1996). TIMI frame count: A quantitative method of assessing coronary artery flow. Circulation.

[20] Jiang Y.T., Yan Z.M., Gu W., Guo H.S., Li X.T., Zheng S.Q., Liao X., Xue D.G. (2025). Advanced Lung Cancer Inflammation Index as a Predictor of Coronary Slow Flow Phenomenon in Patients with Angina and Non-Obstructive Coronary Arteries. Int. J. Gen. Med..

[21] Beltrame J.F., Limaye S.B., Horowitz J.D. (2002). The coronary slow flow phenomenon--a new coronary microvascular disorder. Cardiology.

[22] Alvarez C., Siu H. (2018). Coronary Slow-Flow Phenomenon as an Underrecognized and Treatable Source of Chest Pain: Case Series and Literature Review. J. Investig. Med. High Impact Case Rep..

[23] Wang X., Nie S.P. (2011). The coronary slow flow phenomenon: Characteristics, mechanisms and implications. Cardiovasc. Diagn. Ther..

[24] Yilmaz H., Demir I., Uyar Z. (2008). Clinical and coronary angiographic characteristics of patients with coronary slow flow. Acta Cardiol..

[25] Tambe A.A., Demany M.A., Zimmerman H.A., Mascarenhas E. (1972). Angina pectoris and slow flow velocity of dye in coronary arteries--a new angiographic finding. Am. Heart J..

[26] Sezgin A.T., Barutcu I., Sezgin N., Gullu H., Esen A.M., Acikgoz N., Topal E., Ozdemir R. (2006). Contribution of plasma lipid disturbances to vascular endothelial function in patients with slow coronary flow. Angiology.

[27] Tanriverdi H., Evrengul H., Enli Y., Kuru O., Seleci D., Tanriverdi S., Tuzun N., Kaftan H.A., Karabulut N. (2007). Effect of homocysteine-induced oxidative stress on endothelial function in coronary slow-flow. Cardiology.

[28] Hawkins B.M., Stavrakis S., Rousan T.A., Abu-Fadel M., Schechter E. (2012). Coronary slow flow--prevalence and clinical correlations. Circ. J..

[29] Rafiei A., Ferns G.A., Ahmadi R., Khaledifar A., Rahimzadeh-Fallah T., Mohmmad-Rezaei M., Emami S., Bagheri N. (2021). Expression levels of miR-27a, miR-329, ABCA1, and ABCG1 genes in peripheral blood mononuclear cells and their correlation with serum levels of oxidative stress and hs-CRP in the patients with coronary artery disease. IUBMB Life.

[30] Özmen M., Karakelleoğlu Ş., Ardahanli İ. (2022). Comparison of Ischemia-modified Albumin and Exercise Stress Test in Stable Angina Pectoris. E J. Cardiovasc. Med..

[31] Khalil M.A., Khalfallah M., Elsheikh A. (2024). Predictors and clinical outcomes of slow flow phenomenon in diabetic patients with chronic coronary syndrome. BMC Cardiovasc. Disord..

[32] Binak E., Gunduz H., Sahin M., Kurtoglu N., Dindar I. (2006). The relation between impaired glucose tolerance and slow coronary flow. Int. J. Cardiol..

[33] Kaplan M., Abacioglu O.O., Yavuz F., Kaplan G.I., Topuz M. (2021). Slow Flow Phenomenon Impairs the Prognosis of Coronary Artery Ectasia as Well as Coronary Atherosclerosis. Braz. J. Cardiovasc. Surg..

[34] Akkaya H., Gunturk E.E. (2020). The relationship between coronary slow flow phenomenon and carotid femoral pulse wave velocity and aortic elastic properties. JRSM Cardiovasc. Dis..

[35] İslamoğlu Y., Aksakal E. (2009). Kardiyak Sendrom X Ve Koroner Yavaş Akım. Haseki Tıp Bülteni.

[36] Caliskan M., Erdogan D., Gullu H., Topcu S., Ciftci O., Yildirir A., Muderrisoglu H. (2007). Effects of atorvastatin on coronary flow reserve in patients with slow coronary flow. Clin. Cardiol..

[37] Cakmak M., Tanriverdi H., Cakmak N., Evrengul H., Cetemen S., Kuru O. (2008). Simvastatin may improve myocardial perfusion abnormality in slow coronary flow. Cardiology.

[38] Basta G., Chatzianagnostou K., Paradossi U., Botto N., Del Turco S., Taddei A., Berti S., Mazzone A. (2016). The prognostic impact of objective nutritional indices in elderly patients with ST-elevation myocardial infarction undergoing primary coronary intervention. Int. J. Cardiol..

[39] Wada H., Dohi T., Miyauchi K., Jun S., Endo H., Doi S., Konishi H., Naito R., Tsuboi S., Ogita M. (2018). Relationship between the prognostic nutritional index and long-term clinical outcomes in patients with stable coronary artery disease. J. Cardiol..

[40] Poyraz E., Savas G., Erdem A., Dinc Asarcikli L., Yazici S., Osken A., Guzelburc O., Terzi S. (2022). The Mean Corrected TIMI Frame Count Could Predict Major Adverse Cardiovascular Events in Patients with Coronary Slow-Flow Phenomenon. Turk. Kardiyol. Dern. Ars..

[41] Ford T.J., Ong P., Sechtem U., Beltrame J., Camici P.G., Crea F., Kaski J.C., Bairey Merz C.N., Pepine C.J., Shimokawa H. (2020). Assessment of Vascular Dysfunction in Patients Without Obstructive Coronary Artery Disease: Why, How, and When. JACC Cardiovasc. Interv..

